# Small RNA and Degradome Sequencing Reveal Roles of miRNAs in the Petal Color Fading of *Malus* Crabapple

**DOI:** 10.3390/ijms241411384

**Published:** 2023-07-13

**Authors:** Hao Rong, Xin Han, Yue Xin, Zhouxian Ni, Wangxiang Zhang, Li’an Xu

**Affiliations:** Co-Innovation Center for Sustainable Forestry in Southern China, College of Forestry, Nanjing Forestry University, Nanjing 210037, China; ronghao@njfu.edu.cn (H.R.); hanxin@njfu.edu.cn (X.H.); xiny@njfu.edu.cn (Y.X.); nzhx0627@njfu.edu.cn (Z.N.); malus2011@163.com (W.Z.)

**Keywords:** *Malus* crabapple, small RNA, degradome, miRNA, petal fading

## Abstract

The *Malus* crabapple is an important woody ornamental plant. The fading of petals during its development significantly affects their ornamental value. Petal color is related to anthocyanin content and miRNAs play an important role in the post-transcriptional regulation of anthocyanin synthesis. However, the mechanisms underlying miRNA regulation of petal fading have rarely been studied. Transcriptome and small RNA sequencing of petals from the blooming phases of *Malus*. ‘Indian Summer’ varieties S1 (small bud), S2 (initial-flowering), and S3 (late-flowering) allowed us to identify 230 known miRNAs and 17 novel miRNAs, including 52 differentially expressed miRNAs which targeted 494 genes and formed 823 miRNA–target pairs. Based on the target gene annotation results, miRNA–target pairs were screened that may be involved in the fading process of *Malus* crabapple petals through three different pathways: anthocyanin synthesis, transport, and degradation, involving mcr-miR858-*MYB1*\*MYB5* and mcr-miR396-*McCHI* inhibiting anthocyanin synthesis; mcr-miR167, mcr-miR390, mcr-miR535, and mcr-miR858 inhibiting anthocyanin transport from the cytoplasm to the vacuole by targeting ABC transporter genes (*ABCB*, *ABCC*, *ABCD*, and *ABCG*); and mcr-miR398 targeting the superoxide dismutase genes (*CZSOD2* and *CCS*) to accelerate anthocyanin degradation. These findings offer a novel approach to understanding the mechanism of petal fading and serve as a reference for other plants with floral fading.

## 1. Introduction

Ornamental crabapple is a broad term used for ornamental plants of *Malus* Mill. of the Rosaceae family. Because of its colorful flowers, it is considered an important woody flowering plant in spring. On one hand, the plant’s vibrant flower colors attract insects for pollination, while also increasing the ornamental value of plants. The inhibition of anthocyanin synthesis, transfer processes, and degradation lead to the fading of petals during flower development, thus reducing the ornamental value of plants. This phenomenon has been observed in *Malus* crabapple [1], *Rosa rugosa* [2], *Paeonia lactiflora* [3], *Lycoris longituba* [4], *Silene littorea* [5], chrysanthemum [6], and other plants, but is most evident during the flower opening of ornamental crabapple. Transcriptome and metabolomic analyses have been used to study color fading in plant petals. However, the mechanisms by which miRNAs regulate plant petal fading have rarely been reported.

Anthocyanins are flavonoid pigments that are widely distributed in flowers, fruits, leaves, and other plant tissues. They are synthesized via the phenylalanine pathway by enzymes located on the cytoplasmic face of the endoplasmic reticulum. These enzymes are classified into two groups based on the order in which they are synthesized. Early biosynthetic genes (EBGs), which include phenylalanine ammonia-lyase (PAL), 4-coumaryl: CoA ligase (4CL), chalcone synthase (CHS), chalcone isomerase (CHI), and flavanone 3-hydroxylase (F3H). CHS when expressed as an antisense transgene in *Petunia hybrida* causes anthocyanin accumulation in the petals to be inhibited and the flower color to be pale or even white [7,8]. The late biosynthetic genes (LBGs) of anthocyanin biosynthesis include flavonoid 3′5′ -hydroxylase (F3′5′H), dihydroflavonol 4-reductase (DFR), anthocyanidin synthase (ANS), flavonol-3-glucosyltransferase (3GT), rhamnosyl transferase (RT), anthocyanin acyltransferase (AAC), putative anthocyanin transporter (PAT), and glutathione-S-transferase (GST). In *Gerbera hybrida*, mutations in the *DFR* lead to the inhibition of anthocyanin synthesis and the generation of white flower mutants [9]. ANS can convert colorless leucoanthocyanidins into colored anthocyanins, and the overexpression of peonia *PIANS* and *PIDFR* in tobacco significantly increases anthocyanidin content and leads to the deepening of petal color [10]. ANS deletion or inhibition blocks anthocyanin synthesis in gentian petals, resulting in white or pale petals [11,12]. Previous studies have demonstrated that GSTs play an important role in anthocyanin transport, and *PhAn9* can compensate for the functional loss of maize *BZ2* mutants by participating in anthocyanin transport and accumulation [13]. *DcGSTF2* overexpression in *Dianthus caryophyllus* can increase anthocyanin content, resulting in a deeper petal color [14]. Moreover, overexpression of the *Euphorbia pulcherrima Bract1* gene restored the anthocyanidin-deficient phenotype of the Arabidopsis *tt19* mutant [15]. These genes are closely related to anthocyanin synthesis and changes in their expression patterns can affect anthocyanin synthesis.

Anthocyanin biosynthesis is regulated by MYB, bHLH, WD40, and other transcription factors [16,17], with R2R3-MYB being the most critical. *AtMYB75*, *AtMYB90*, *AtMYB113*, *AtMYB114* of *Arabidopsis thaliana* [18], and *PhAN2* of *P. hybrida* [19] promote anthocyanin synthesis. Transcriptome analysis of the petals of *Malus halliana* revealed that *MhMYB10* was significantly downregulated during flower development and positively correlated with anthocyanin content. Further analysis has shown that methylation of the *MhMYB10* promoter region inhibits its expression and may be involved in petal fading [1]. Certain R2R3-MYB transcription factors, such as *AtMYB4*, *PhMYB4*, *PhMYB27*, and *MdMYB16*, which negatively regulated anthocyanin biosynthesis, lost their ability to bind to target gene promoters but retained their ability to bind bHLH. They compete with the positively regulated R2R3-MYB and thus play a negative regulatory role in anthocyanin biosynthesis [20,21,22,23].

Compared to anthocyanin synthesis and transport, anthocyanin degradation in plants has received less attention and focus. The degradation of anthocyanins depends on the oxidation reaction of active enzymes. Whether this is independent of the oxidation reaction of flower senescence or a branch of the oxidation reaction of aging was not revealed until 2005 by VAKNIN et al. Studies on the degradation of *Brunfelsia calycina* anthocyanin reported that β-glucosidase involved in anthocyanin degradation was newly synthesized, and the process was much earlier than the process of flower senescence. Inhibition of β-glucosidase activity by D-gluconic acid significantly reduces anthocyanin degradation [24]. Additionally, peroxidase PPOs and β-glucosidase are also involved in the degradation of anthocyanin in wine and juice [24,25].

miRNAs are a class of endogenous non-coding small single-stranded RNAs that are widely present in eukaryotes, whose length is generally between 18–25 nt, which play an important role in secondary metabolism. As important regulatory factors in plants, miRNAs regulate the accumulation of metabolites by directly degrading the genes related to metabolic pathways. Studies on miR156, miR828, and miR858 have shown that miRNAs regulate anthocyanin synthesis at the post-transcriptional level. miR156 targets *AtSPL9*, which can competitively bind *PAP1* with *TT8*, affects the stability of the MYB-bHLH-WD40 transcription complex and directly prevents the expression of anthocyanin biosynthesis genes [26]. The heterologous expression of miR156e-3p in peony in Arabidopsis resulted in the decreased expression of SPL transcription factors encoding negative regulators of anthocyanin accumulation, but the strong expression of DFR promoted the accumulation of lateral anthocyanins in Arabidopsis [27]. Utilizing the pre-miR156a sequence of the ‘Golden Delicious’ apple, Zhang et al. (2020) cloned miR156a from *Malus* crabapple via homologous cloning, and analyzed its expression levels at four different developmental stages [28], but did not conduct further studies. Although studies on the regulation of anthocyanins by miRNAs have been reported for many plants, there is still a lack of studies on the color of ornamental crabapples. Systematic studies on miRNA identification during the crabapple petal fading process and miRNA–target regulatory networks have not been reported.

In this study, transcriptome, small RNA, and degradome sequencing were conducted on petals at three different developmental stages of *Malus* ‘Indian Summer,’ to explore the miRNA related to the fading process, and the miRNA–target regulatory network during the fading process was constructed via association analysis. These results provide a new idea for studying the fading mechanism of ornamental petals and a reference for other plants with flower fading.

## 2. Results

### 2.1. Reference Transcriptome Assembly

Using strand-specific RNA libraries rather than NEB allows for more accurate gene quantitation, localization, and annotation information as well as translation transcripts and single-exon expression levels in each isoform. Transcriptome sequencing yielded 69,581,194 raw reads, with 68,906,804 clean reads (10.34 GB) obtained after strict quality control. The data had an overall error rate of 0.03%. That of Q20 and Q30 were 97.92% and 93.97%, respectively, and their GC content was 46.92%. After assembly and comparison with the reference genome, high-quality reference transcriptome data were obtained for miRNA and target gene prediction.

### 2.2. Annotation of Small RNA and Prediction of Novel miRNA

In total, 5.398 G of data was obtained from nine small RNA libraries of *M*. ‘Indian Summer’ flower at three different development stages (S1-1, S1-2, and S1-3; S2-1, S2-2, and S2-3; S3-1, S3-2, and S3-3). An average of 0.60 G of raw reads was obtained from each small RNA library. By removing low-quality reads, reads with an N (N indicates that base information cannot be determined) ratio greater than 10%, reads contaminated by a 5′ adapter, reads without a 3′ adapter sequence and inserted fragments, and poly A/T/G/C reads, high-quality clean reads were obtained for subsequent analysis (Table 1).

**Figure 1:**
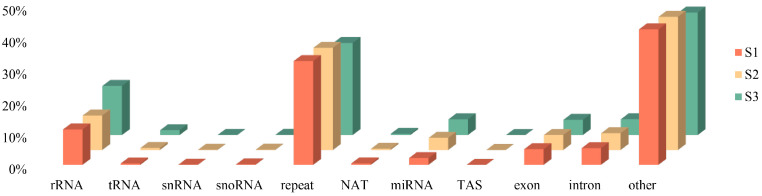
Classification and abundance of sRNA in S1, S2, and S3.

In general, the lengths of sRNA in plants were between 18 and 30 nt. Accordingly, we screened the clean reads again, and 97,406,413 reads in the 2.228 Gb range were obtained (Table 2). The positioning results of sRNA obtained with the Bowtie2-2.1.0 software revealed that 81.33–84.57% of mismatched sRNA could be mapped to the reference sequence, 55.26–59.57% could be mapped to the positive-sense strand of the reference sequence, and the rest could be mapped to the antisense strand (Table 3). Furthermore, all alignments of sRNAs on the reference sequences were classified and annotated (Figure 1). Repeated sequences accounted for the highest proportion (approximately 31.3%, on average). These repeated sequences may bind to different Argonaute proteins to participate in important biological processes, such as DNA methylation and transposon regulation. In contrast, the number of sRNAs containing miRNAs accounted for only 2.13% (S1), 3.81% (S2), and 4.96% (S3), suggesting that miRNAs are involved in regulating flower development and the fading process.

**Figure 2:**
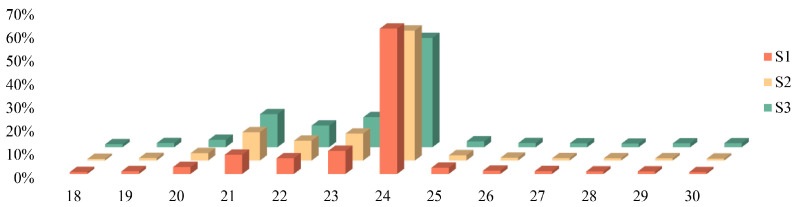
Distribution of small RNA length in S1, S2, and S3.

The statistics of the length and quantity of sRNA obtained demonstrated that the characteristic distribution of sRNA in nine samples at the three stages was similar, and the quantity of sRNA at different lengths was significantly different, among which 24 nt sRNAs were the most abundant, accounting for 62.15%, 55.60%, and 46.71% in the three different developmental stages, respectively, followed by 23 nt and 21 nt (Figure 2).

### 2.3. Identification of Known miRNA and Novel miRNA

A total of 3614 unique sRNAs were obtained using the miRDeep-P2-v1.1.4 and mirDeep2.0.1.2 software to analyze 2,946,473 sRNA sequences containing miRNAs by removing inaccurate Dicer shear sites (miRNA/miRNA* overhang of 1< or >3 nt, as well as miRNA expression of <10). According to the RNAfold prediction, 194 pre-miRNAs containing hairpin structures were obtained, corresponding to 247 miRNAs (Table 4, Table S2). Finally, the miRbase22.0 database was searched (*E*-value < 10), and 230 known miRNAs were retrieved under the conditions of a mismatch of <4 and a gap of <2.

**Figure 3:**
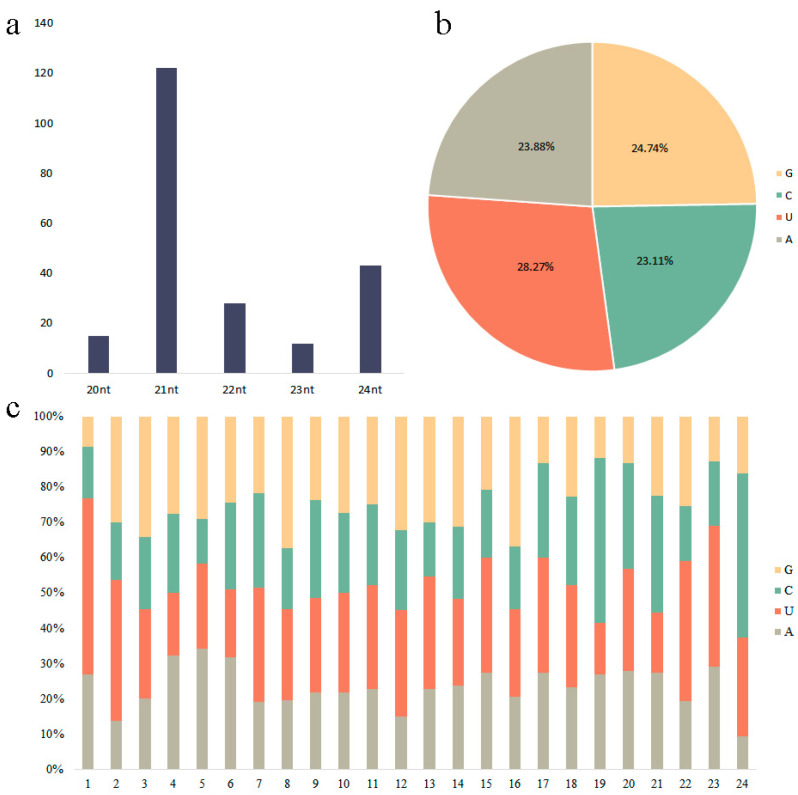
The length profile of the known miRNAs and the sequence characteristics. (**a**) Base preference analysis of known miRNA; the x-axis represents the length of known miRNA, and the y-axis represents the corresponding number of known miRNAs. (**b**) Base distribution of known miRNAs. (**c**) Statistical distribution of sequence length of known miRNAs; the x-axis represents position, and the y-axis represents the percentage of the base composition.

Among the 230 known miRNAs, 128 miRNAs with a length of 21 nt were the largest, followed by 24 nt with 43, 20 nt, 22 nt, and 23 nt with 16, 31, and 12 nt, respectively. Among the known miRNAs, the proportion of the four bases was the highest in U (28.27%), followed by G (24.74%), A (23.88%), and C (23.11%), all with a relatively uniform distribution. The results indicated that the first base at 5′ had the highest frequency towards U but the highest resistance to G among 230 known miRNAs, which was consistent with the preference of the first base in plant miRNAs (Figure 3).

**Figure 4:**
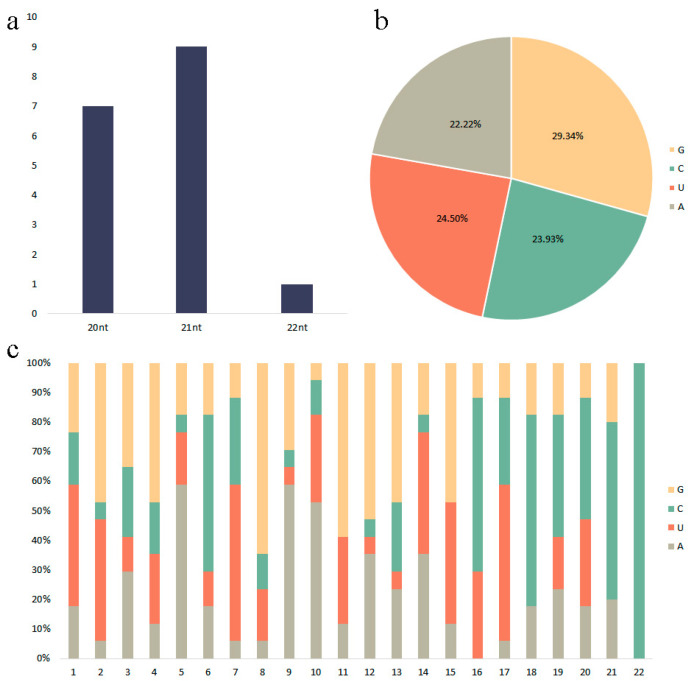
The length profile of the novel miRNAs and the sequence characteristics. (**a**) Base preference analysis of novel miRNA; the x-axis represents the length of novel miRNA, and the y-axis represents the corresponding number of novel miRNAs. (**b**) Base distribution of novel miRNA. (**c**) Statistical distribution of sequence length of novel miRNA; the x-axis represents position, and the y-axis represents the percentage of the base composition.

The novel miRNA prediction method was the same as that used for known miRNAs. After comparison in miRbase 22.1, 17 novel miRNAs of *Malus* crabapple were predicted, including five novel miRNAs and 12 novel miRNAs*. The lengths of these novel miRNAs ranged from 20 to 22 nt, among which the number of miRNAs with a length of 21 nt was the largest (nine), and the number of miRNAs with a length of 20 and 22 nt was seven and one, respectively. This result was similar to the length distribution of apple miRNAs in the miRBase 22.1 databases. All miRNAs with a length of 21 nt were dominant, while no miRNAs with lengths of 23 nt and 24nt were found.In terms of the distribution of A, U, G, and C bases, G occupies the highest proportion (29.34%), followed by U (24.50%), C (23.93%), and A (22.22%). In addition, the first base at the 5′ end of novel miRNAs had a strong base preference for U, which was consistent with the preference of the first base in plant miRNAs (Figure 4).

### 2.4. miRNA Expression Profile and Identification of Differentially Expressed miRNAs

**Figure 5:**
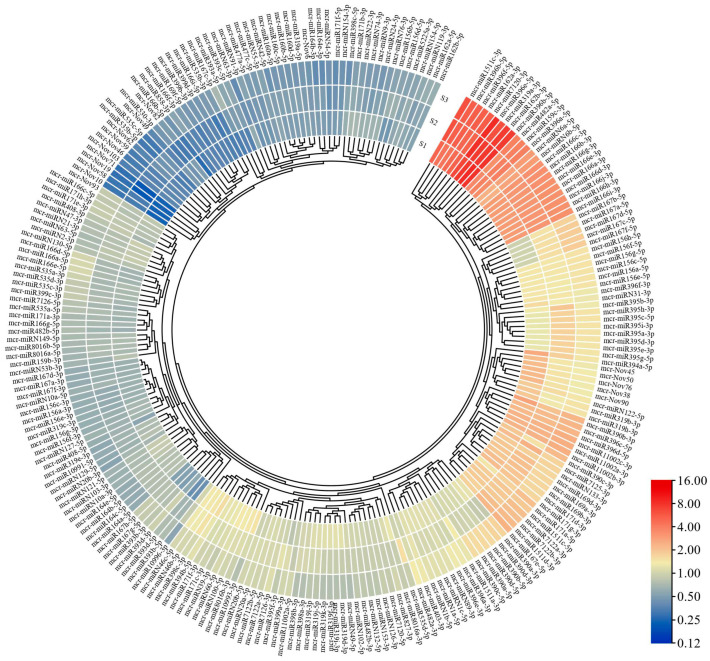
miRNA expression calorimetry map. The expression profiles of all miRNAs in S1, S2, and S3. Abundance is demonstrated with a color gradient by normalized log2-transformed values. Blue indicates low expression, and red indicates high expression.

To determine the expression levels of miRNAs in the three different developmental stages, we created a heat map (Figure 5) after normalizing the miRNA expression levels in each sample. A total of 23,102 and 112 miRNAs were classified as high-, medium-, and low-abundance according to TPM (Table S3). The expression levels of nine miRNAs, including mcr-miR1511c-3p, mcr-miR396b-5p, and mcr-miR396b-3p, were the highest.

**Figure 6:**
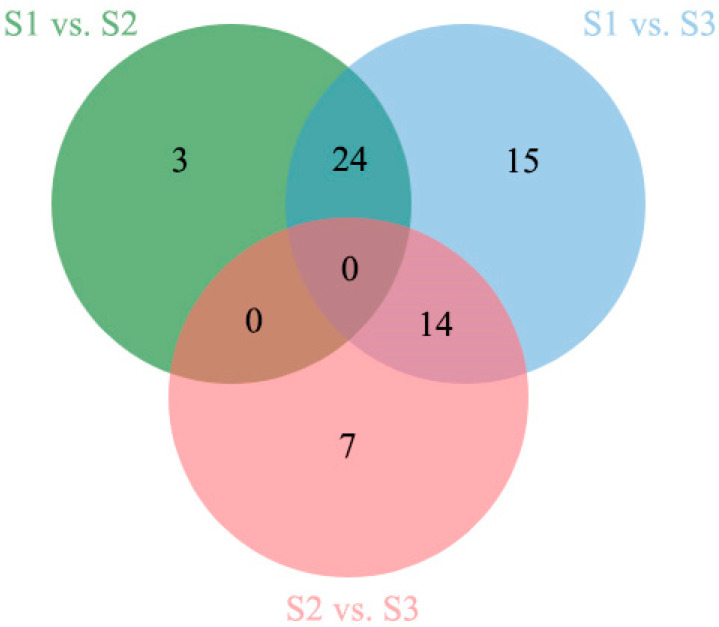
Number of DEMs (differential expression miRNAs) in S1, S2, and S3.

To explore the expression trend of miRNAs in the flower fading process, we found that 62 miRNAs were differentially expressed in the S1, S2, and S3 stages, among which 35 miRNAs were upregulated and 28 were downregulated (Table S4). According to the results of the differential expression of miRNA (Figure 6), there were 26 differentially expressed miRNAs in S1 vs. S2, 39 differentially expressed miRNAs in S1 vs. S3, and 20 differentially expressed miRNAs in S2 vs. S3. There were 24 differentially expressed miRNAs shared by S1 vs. S2 and S1 vs. S3, and the numbers of upregulated and downregulated miRNAs were consistent (12 in both cases). The results showed that miRNAs actively respond to the developmental process of flowers, and different miRNA responses may differ at different developmental stages, suggesting that miRNAs play an important role in regulating the fading process of flowers.

**Figure 7:**
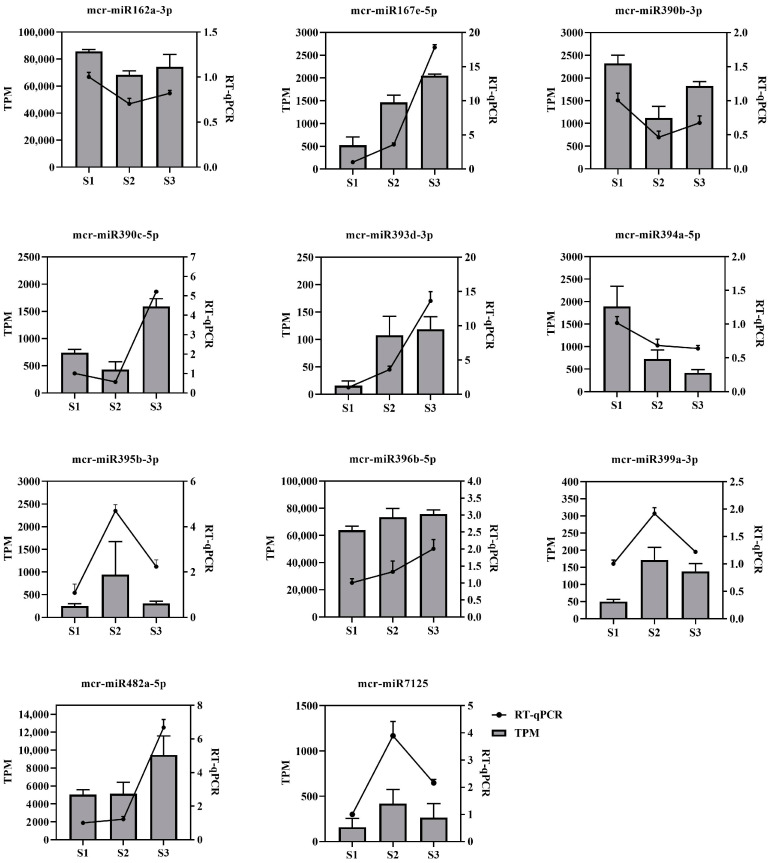
Validation of miRNA quantification. Comparison and correlation analysis of 11 miRNA expression profiles. The x-axis represents three stages of the flower: S1, S2, and S3. The y-axis represents the relative expression level and TPM (transcripts per million).

To verify the accuracy of the miRNA sequencing results, a total of 11 miRNAs were randomly selected from DEMs for real-time quantitative PCR (RT-qPCR) analysis. By comparing the RT-qPCR results with the TPM results, it was found that the expression trend of these miRNAs between RT-qPCR and TPM was consistent, which proved the reliability of the sequencing results (Figure 7).

### 2.5. Target Gene Prediction and Functional Enrichment Based on Degradome Sequencing

To reduce the number of false-positive target genes, the degradome sequencing results were used to remove false-positive targets without mRNA cutting sites. A total of 22,232,259 raw reads and 8,699,925 unique raw reads were obtained via the degradome sequencing. Remove short reads (<15 nt) and 3′ adaptors, 8,660,707 unique reads were mapped to 4,997,426 unique reads, which covered 29,591 transcripts (66.23% of 44,677 reference transcripts) (Table 5). The target genes were predicted using CleaveLand v4.0, and miRNA–target pairs were divided into zero, one, two, three and four types according to DegradomeCategory, and the third and fourth types were eliminated to improve prediction accuracy. Finally, 1641 target genes of 194 miRNAs were obtained, resulting in 3971 putative miRNA–target pairs, of which there were 300, 80, and 3591 target plots of types 0, 1, and 2, respectively. Target plots (T-plots) of these miRNA–target pairs were plotted (https://figshare.com/articles/dataset/The_T-plot_of_all_the_miRNA-target_pairs/23592387, accessed on 28 June 2023). Functional annotation identified 172 transcription factors (TFs), 30 transcription regulators (TRs), 82 kinases, and 211 other genes and predicted putative proteins in 1148 reference genomes among the 1641 target genes. 

**Figure 8:**
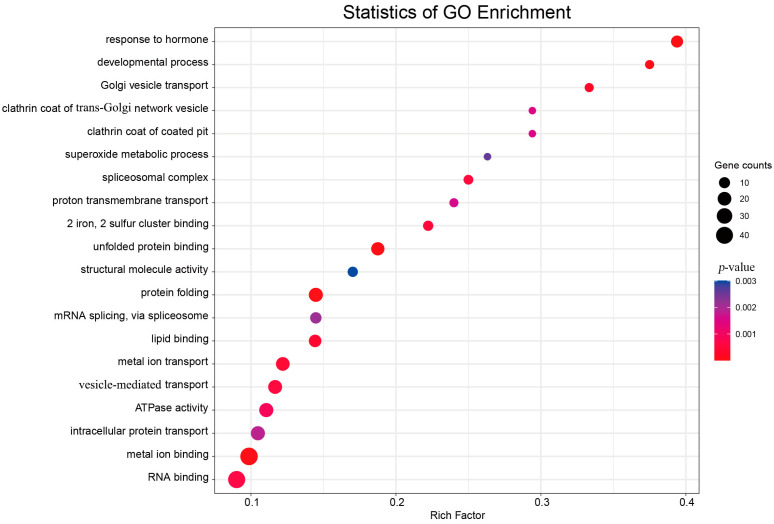
Scatter diagram of target gene GO–terms pathway. Gene counts represent the total number of target genes in the pathway; the *p*-value was obtained via a hypergeometric test. The left coordinate represents the path name, and the horizontal coordinate represents the enrichment factor.

GO enrichment analysis of the target genes demonstrated that 1577 target genes were annotated to 436 terms for three parts: biological process (BP), molecular function (MF), and cellular component (CC). Intracellular protein transport (GO:0006886), protein folding (GO:0006355) in BP, intracellular protein transport (GO:0005622) in CC, and terms such as metal ion transport (GO:0046872) and RNA binding (GO:0003723) in MF were significantly enriched (Figure 8). The plant GO model of agriGO v2.0 [29] was used for the highly interconnected GO clustering of DEM target genes. The nucleus in CC (GO:0005634), protein binding in MF (GO:0005515), regulation of DNA-templated transcription (GO:0006355), and DNA-templated transcription (GO:0006351) in BP were significantly activated during petal fading.

**Figure 9:**
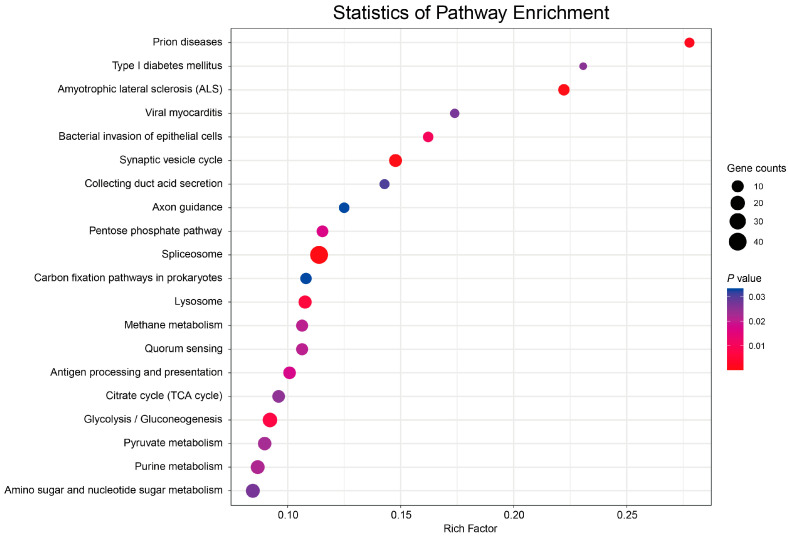
Scatter diagram of KEGG pathway of target gene. Gene counts represent the total number of target genes in the pathway; the *p*-value was obtained via a hypergeometric test. The left coordinate represents the path name, and the horizontal coordinate represents the enrichment factor.

The KEGG metabolic pathway analysis of target genes can be classified into metabolism, genetic information processing, environmental information processing, and cellular. There are five major types of processes and organismal systems involved in 287 KEGG metabolic pathways. Among them, spliceosomes (ko03040), protein processing in the endoplasmic reticulum (ko04141), ribosomes (ko03010), glycolysis/gluconeogenesis (ko00010), and plant hormone signal transduction (ko04075) were significantly enriched (Figure 9). In addition, enrichment analysis of DEMs target genes showed that plant hormone signal transduction (ko04075), spliceosome (ko03040), pyruvate metabolism (ko00620), glycolysis/gluconeogenesis (ko00010), and the citrate cycle (TCA cycle) (ko00020) were significantly enriched. Additionally, pathways involved in anthocyanin synthesis and transport were significantly enriched.

### 2.6. miRNA Regulatory Network and Key Components in the Petal Fading Process

**Figure 10:**
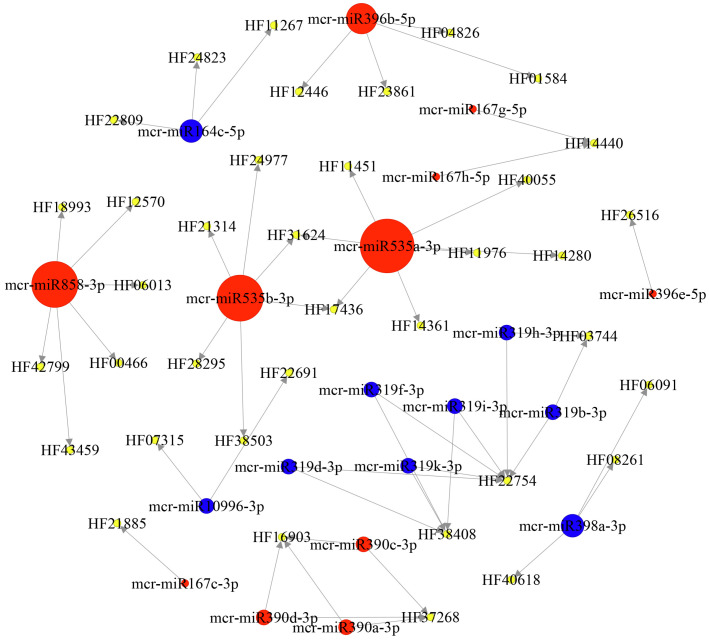
DEM regulatory network diagram. Red nodes indicate upregulated miRNA, blue nodes indicate downregulated miRNA, and yellow nodes indicate target genes.

The construction of a DEM–target regulatory network plays an important role in studying the molecular mechanisms underlying the post-transcriptional regulation of flower fading in ornamental crabapples. Among the 494 target genes, regulatory network analysis showed that 52 of the 61 DEMs targeted 494 genes, forming 823 miRNA–target pairs. Functional annotation identified 69 transcription factors, 20 kinases, 69 other genes, and 336 predictive proteins. To further study the DEM–target gene network regulating petal fading, we screened 37 genes that were annotated to the MYB, bHLH, WRKY, and other transcription factors, phenylpropanoid biosynthesis (ko00940), ABC transporter (ko02010), and peroxisome (ko04146) pathways, which were targeted by 20 miRNAs from nine families (Figure 10, Table S5). For example, two MYB transcription factor genes, HF00466 (*MYB1*) and HF18993 (*MYB5*), as well as HF06013 (*ABC* transporter-like) were targeted by mcr-miR858. HF23861 (*McCHI*) was targeted by mcr-miR396b-5p. The ABC transporter gene HF14440 (*ABCC*) was targeted by mcr-miR167g-5p and mcr-miR167h-5p. Similarly, HF37268 (*ABCB*) was targeted by mcr-miR390a-3p, mcr-miR390c-3p, and mcr-miR390d-3p. Additionally, mcr-miR535a-3p targeted the ABC transporter gene HF14280 (*ABCD*), while mcr-miR535b-3p targeted HF24977 (*ABCG*). Additionally, mcr-miR398a-3p targeting three genes were annotated to the peroxisome (ko04146) pathway including HF06091 (*CZSOD2*), HF08261 (*CCS*) and HF40618 (*CZSOD2*), all of them belong to the superoxide dismutase family. These miRNA–target pairs might inhibit anthocyanin synthesis and transportation, and accelerate the anthocyanin degradation rate, thus directly or indirectly leading to the fading of petals.

## 3. Discussion

Flower fading throughout development has a significant impact on the ornamental value of this essential woody ornamental plant in the temperate zones of the Northern Hemisphere. Therefore, it is critical to investigate the fading of petals and reveal the underlying molecular regulatory mechanisms. miRNAs are important regulatory factors in plants that regulate gene expression at the post-transcriptional level and play an important role in regulating anthocyanin synthesis in flowers and fruits [27,30,31]. Petal fading is the opposite phenomenon of flower and fruit coloration and is associated with anthocyanin synthesis, transport obstruction, and anthocyanin degradation. However, it is uncertain whether or not this process is regulated by miRNAs in the same manner as that of miRNAs during coloration. As a result, in this study, small RNA sequencing was performed on the petals of ornamental crabapples at different developmental stages to identify miRNAs involved in the fading process, and combined with degradome sequencing, miRNA target genes involved in the regulation network of petal fading were constructed, providing a basis for further research on the fading of ornamental crabapple petals.

### 3.1. Analysis of miRNA Sequence Characteristics during Petal Fading

With the development of high-throughput sequencing technology, small RNA sequencing has become an important tool for miRNA discovery, and is widely used to identify miRNAs in many plants. Yu et al. identified 31 potential miRNAs from 16 families from 324,000 apple EST sequences and verified 16 of them using miR-RACE technology [32]. Ye et al. used bioinformatics to predict 154 conserved miRNAs in the apple genome [33]. Using deep sequencing on apple small RNA, Xia et al. identified 75 miRNAs, among which 42 apple-specific miRNAs exhibited different expression patterns [34]. Currently, 322 miRNAs from apples are included in the miRBase database. Although considered an important ornamental plant in the *Malus* spp., ornamental crabapples still lack relevant reports on miRNA identification and functional studies.

In this study, during the development of ornamental crabapple flowers, high-throughput small RNA sequencing was performed on the petals of S1, S2, and S3. In total, 247 miRNAs were identified, including 230 known and 17 novel miRNAs. According to the miRNA length statistics, both the known and novel miRNAs had the largest number of 21 nt, and the length of apple miRNA in the miRBase database was also mainly 21 nt, indicating that the length characteristics of miRNAs of ornamental crabapple were consistent with the overall characteristics of apples. The first base preference analysis showed that both novel miRNAs and known first ribonucleic acid bases have a strong preference for U and some resistance to G, which is consistent with the behavior of most plants. This bias is attributed to the stability of RISC, the recognition pattern of the AGO protein for the 5′ nucleotide of miRNA, and the specificity of the cleavage site [35,36], proving the accuracy of sequencing and prediction results.

### 3.2. Target Gene Prediction

miRNA-mediated mRNA splicing is the main mechanism by which miRNAs function in plants; therefore, the identification of target genes is an important step in the study of miRNA function. Degradome sequencing technology has been widely used to predict miRNA target genes in plants owing to its high efficiency, speed, accuracy, and high throughput. In this study, 1641 target genes of 193 miRNAs (247 in total) were predicted via degradome sequencing, and 3972 putative miRNA–target pairs were identified. The 494 target genes of the 52 DEMs (61 in total) were predicted to form 823 miRNA–target pairs. In addition, the target genes of the 54 identified expressed miRNAs were not detected via degradome sequencing; therefore, we speculated that these 54 miRNAs might exert their functions through translation inhibition.

### 3.3. miRNA Targeting Anthocyanin Biosynthesis Is Involved in Petal Fading

During flower development, the petal color switches from light to dark or from dark to light, affecting the attractiveness of pollinators [25]. However, the color of the petals changes from dark to light (fading), thus seriously affecting the ornamental value of the plants. Studies have shown that anthocyanin biosynthesis is related to petal color changes. Han et al. used HPLC-DAD to determine the anthocyanin content in the petals of S1, S2, and S3 of *Malus hupehensis* and found that cyanidin-3-galactoside was the most important pigment, which gradually decreased during floral development. The expression levels of the PAL, CHS, CHI, DFR, and ANS gene related to anthocyanin biosynthesis were consistent with the changes in anthocyanin content. During the development of *Malus halliana* flowers, the floral fading phenomenon is also attributed to the expression levels of anthocyanin biosynthesis-related genes, and the decrease in their expression may be affected by the methylation of the *MYB10* promoter region [1,37]. As important regulatory factors, miRNAs are widely involved in the synthesis of secondary metabolites, and their involvement in the regulation of anthocyanin synthesis has been revealed in recent years. In the results of this study, the upregulated expression of mcr-miR858 targets two MYB transcription factors, *MYB1* (HF00466) and *MYB5* (HF18993), where MYB1 is a positive regulator of anthocyanin synthesis of bayberry and blueberry [38,39]. MYB5 positively regulates the accumulation of pigments and proanthocyanidins during biosynthesis [40]. Moreover, miR156-*SPL9* and miR858-*MYBL2* are positive regulatory factors that promote the synthesis and accumulation of anthocyanins in *Arabidopsis thaliana* [26,41]. *Brassica rapa* BrmiR828-*BrPAP1*, *BrMYB82*, and *BrTAS4*, apple miR172-*MYB10*, apple mdm-miR858-*MdMYB9*, and Md*MYBPA1* inhibit anthocyanin biosynthesis [31,42,43]. Therefore, the relationship between miRNAs and anthocyanin synthesis depends on the function of their target genes. Therefore, we speculated that with the opening of flowers, the gradually increased expression of mcr-miR858-3p may inhibit the expression of *MYB1* and *MYB5*, thus inhibiting anthocyanin synthesis and, consequently, the fading of crabapple petals.

Many studies have reported that miRNAs not only participate in anthocyanin synthesis by targeting transcription factors but also regulate anthocyanin synthesis by targeting structural genes involved in anthocyanin biosynthesis. Novel_miR_138 targets *PsCHI* in *Paeonia suffruticosa* [44] and miR168 targets *CHS* in *Canna* [45]; miR2616 and novel-miR25 target F3GT and F3GT7 in peony [46], respectively. These target genes are the key structural genes involved in anthocyanin synthesis. Inhibition of or interference with their expression can directly affect anthocyanin synthesis. The upregulated expression of *McCHI* (HF23861), an important structural gene in anthocyanin synthesis targeted by mcr-miR396b-5p, was found in the following quantitative analysis. *McCHI* expression was the highest in S1 but decreased in S3, which is complementary to the expression trend of mcr-miR396b-5p. mcr-miR396b-5p- *McCHI* inhibits anthocyanin synthesis and plays a key role in flower petal fading. *MdCCR* (cinnamoyl-coenzyme A reductase gene) was targeted by miR7125, thus inhibiting lignin synthesis and inducing anthocyanin accumulation in apples [47]. In this study, DEM mcr-miR10996-3p targeted cinnamate dehydrogenase *McCAD1* (HF22691), which plays an important role in catalytic lignin synthesis [48]. mcR-mir10996-3p-Mc*CAD1* may affect anthocyanin synthesis by competing with anthocyanins as a substrate, causing the petals to fade.

### 3.4. miRNA-Targeting Transporters Are Involved in Petal Fading

After anthocyanins are synthesized in the cytoplasm, they must be transferred to vacuoles for storage and color formation. The efficiency of anthocyanin transport in plants has an important effect on plant color to a large extent [49]. In the analysis of DEMs, the upregulated expression of miRNAs of the mcr-miR167, mcr-miR390, mcr-miR535, and mcr-miR858 families targeting ABC transporters included *ABCC* (HF14440), *ABCB* (HF37268), *ABCD* (HF14280), *ABCG* (HF24977), and HF06013. There are four main proteins involved in anthocyanin transport: glutathione transferase (GSTs), multi-drug resistance-related protein (MRP), multidrug and toxic compound excretion (MATE), and BTL-homologue of bilirubin translocation enzyme [50,51,52,53]. Among these, GSTs participate in anthocyanin transport by conjugating to ABC transporters. For example, *AtABCC2* and *Vitis vinifera* VvABCC1 participate in anthocyanin vacuolar transport by conjugating with GSH [54,55]. In this study, the upregulation of miRNAs inhibited the expression of ABC transporters, thereby affecting the formation of conjugates with GSTs, which inhibited anthocyanin transport to the vacuoles and participated in petal fading.

### 3.5. miRNA Is Involved in Anthocyanin Degradation, Leading to Petal Fading

The decrease in anthocyanin content is the main reason for petal fading. This process is related to anthocyanin biosynthesis and transport; anthocyanin degradation is another important factor. Compared to synthesis, there are few studies on anthocyanin degradation. Vaknin et al. were inspired by the results of anthocyanin degradation in wine and fruit juice and found that there was a significant increase in peroxidase activity during anthocyanin degradation in *Brunfelsia calycina*, which was independent of petal aging [24]. During the development of petals, the downregulated expression of mcr-miR398a-3p resulted in increased expression levels of three superoxide dismutase family genes, HF06091 (*CZSOD2*), HF08261 (*CCS*) and HF40618 (*CZSOD2*), in the peroxisome pathway, which accelerated the degradation of anthocyanins and may be a factor leading to the fading of ornamental crabapple petals. However, further experimental verification is required. Nevertheless, these results provide an important reference for studying the miRNAs involved in anthocyanin degradation.

## 4. Materials and Methods

### 4.1. Plant Materials

**Figure 11:**
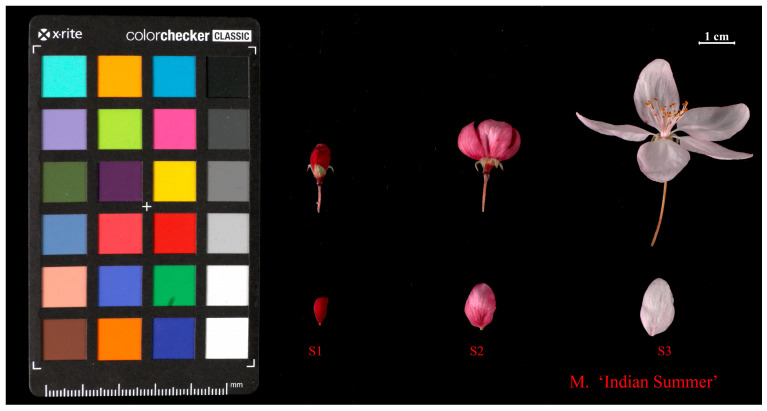
The flower at different developmental stages of *M*. ‘Indian Summer’. S1 (small bud), S2 (initial flowering), and S3 (late flowering).

The materials used in the experiment were obtained from the national repository of *Malus* spp. germplasm, which is located in Jiangdu District, Yangzhou City, Jiangsu Province (119°55′ E, 32°42′ N), China. Through the phenological observation of 105 varieties in the resource nursery, the cultivar *M*. ‘Indian Summer’ whose petals faded obviously during the development stages was selected as the research object. *M*. ‘Indian Summer’ was bred by Robert Simpson at the Simpson Nursery in the United States and has been reported in the International Ornamental Crabapple Society Bulletin [56]. The phenotypic characteristics of this variety can be found on the website (https://www.malusregister.org/cn, accessed on 28 June 2023), where it is described as deep pink buds and pink petals. Based on the relevant study [1], petal samples were collected from S1 (small bud, deep), S2 (initial flowering, light), and S3 (late flowering, lightest) in April 2021 (Figure 11). Three clones of this variety were selected from each period as biological replicates. Nine petal samples were placed in liquid nitrogen and stored at −80 °C. Total RNA from each sample was isolated in accordance with a slightly improved CTAB-LiCl protocol [57] and agarose gel electrophoresis was used to detect the degree of RNA degradation and contamination. In addition, an Agilent Bioanalyzer 2100 system (Agilent Technologies, Santa Clara, CA, USA) was used for quality control, and samples with an RNA integrity index (RIN) of ≥7 were used for subsequent analysis.

### 4.2. Stand-Specific Sequencing for Reference Transcriptome

Stand-specific RNA libraries were constructed with the total RNA of the nine samples using NEBNext^®^ Ultra™ Directional RNA Library ep Kit for Illumina^®^ (NEB, Ipswich, MA, USA), in accordance with the manufacturer’s instructions. Subsequently, Agilent 2100 Bioanalyzer System was used to determine the library insert size. After qualified library testing, Illumina Novaseq 6000 (Illumina, San Diego, CA, USA) was used for high-throughput sequencing. Clean reads were obtained by removing low-quality reads, the reads containing poly-N and 5′ adaptors, or the reads without 3′ adaptors [58]. The clean reads were compared with the apple reference genome (https://github.com/moold/Genome-data-of-Hanfuapple, accessed on 28 June 2023) [59] to obtain a standard reference transcriptome.

### 4.3. Small RNA Sequencing and miRNA Identification

The NEBNext Multiplex Small RNA Library Prep Set for Illumina^®^ (NEB, USA) was used to construct sRNA libraries for the nine samples. LongAmp Taq 2X Master Mix, SR Primer from Illumina, and index (X) primers were used for PCR amplification. The PCR products were purified and recovered on 8% polyacrylamide gel (100 V, 80 min). Library quality was assessed on Agilent Bioanalyzer 2100 System using DNA High Sensitivity Chips and the NovaSeq 6000 platform was used for sequencing. Library construction and high-throughput sequencing were performed by NovoGene Co., Ltd., Beijing, China. Clean reads were obtained after the quality control of raw reads and compared with a standard-specific reference transcriptome to analyze sRNA distribution and classification using the Bowtie2-2.1.0 software [60] at a 0 mismatch base. Small RNAs from mRNA exons, introns, degraded fragments, protein-coding genes, repeat sequences, rRNA, tRNA, snRNAs, and snoRNAs were excluded to improve the accuracy of miRNA prediction.

### 4.4. Identification, Quantification, and Differential Expression Analysis of miRNA

To avoid a high false positive rate in direct miRBase prediction, miRNAs were identified via prediction and then compared (Figure S1). The first step was the prediction of the pre-miRNAs. miRDeep-P2-v1.1.4 [61] and mirDeep2.0.1.2 [62] were used to predict the secondary structure of the tag sequences containing sRNA, the Dicer cleavage site, and the minimum free energy. In addition, the number and distribution of pre-miRNA tags were determined and non-conforming pre-miRNA tags were removed. Then, according to the annotation standards of plant miRNAs, the pre-miRNAs were manually proofread [63]. Finally, the sRNA tags containing pre-miRNA secondary structures, miRNAs, and miRNAs* were obtained. RNAfold software (http://rna.tbi.univie.ac.at/cgi-bin/RNAWebSuite/RNAfold.cgi, accessed on 28 June 2023) was used to predict the hairpin RNA sequences in the second step. The miRNAs and miRNA* were compared with plant miRNAs in the miRbase22.0 database (http://www.mirbase.org/, accessed on 28 June 2023) [64]. The known miRNAs and pre-miRNAs were classified and named for comparison, and the remaining miRNAs were considered novel miRNAs. Finally, the miDeep2 quantifier.pl was used to obtain the length, base bias on the first position, and base on each position of all the identified miRNAs.

miRNA expression levels were estimated via transcripts per million (TPM). Differentially expressed miRNAs (DEMs) were identified using the DESeq R package (version 1.24.0) with adjusted *p*-values using the Benjamini and Hochberg methods. A corrected *p*-value of <0.05 was set as the threshold for screening differentially expressed genes.

Total RNA was extracted from petals in accordance with the method described by Huang et al. (2020) [65]. DEMs were verified using RT-qPCR, and their expression patterns were analyzed. Reverse transcription of miRNAs was performed using Mir-XTM miRNA First-Strand Synthesis and TB Green RT-qPCR (Takara Bio, Inc., Mountain View, CA, USA). Specific primers and the universal primer mRQ3 were used for the RT-qPCR analysis. Specific primers for miRNA RT-qPCR are listed in Table S1, and all primers were synthesized by GenScript Biotechnology Co., Ltd., Nanjing, China. The reaction was performed in accordance with the instructions of PowerUp SYBR Green Master Mix (ABI, Carlsbad, CA, USA). The reference gene, which was selected from small RNA sequencing, was used as a control for normalization with the 2^−ΔΔCt^ method.

### 4.5. Prediction and Screening of Target Genes via Degradome Sequencing

Poly(A) RNA was purified from total plant RNA (20 μg) using poly T oligo-attached magnetic beads after two rounds of purification. The 5′ adapters were ligated to the 5′ end of the 3′ cleaved mRNA fragments from miRNA-induced cleavage by RNA ligase. Biotinylated random primers and mRNA were mixed for reverse transcription and amplified via PCR to construct a cDNA library with an average insert size of 200–400 bp. Finally, we performed 50 bp single-end sequencing on Illumina HiSeq 2500 (LC Bio, Hangzhou, China) following the vendor’s recommended protocol.

Clean reads were used to validate the miRNA–target pairs after quality control and noncoding RNA removal. Targets were predicted using CleaveLand v4.3 program [66] and Oligomap was used to map the degradome data to the reference transcriptome and construct a degradome density file. Standard sequences that were valuable for the degradome density files were compared in the NRPM database (per million reads) to remove redundancy. Target genes paired with miRNA sequences were predicted using GSTAr. Finally, the results of the two software programs were integrated to determine the common mRNA that was the target of the miRNA. The T-plot of the miRNA–target pairs was plotted based on degradome density files. Based on the abundance of reads at the cleavage site (RCSs), the miRNA targets were divided into five categories from high to low reliability: 0, 1, 2, 3, and 4.

### 4.6. Target Gene Function Enrichment

Gene ontology (GO) and KEGG enrichment analysis were used on the target gene candidates of DEMs to investigate the biological significance of miRNA. The GOseq R package was used to realize the Wallenius non-central hypergeometric distribution for the GO enrichment analysis. KOBAS software (version 2.0) was used to test the statistical enrichment of target genes in the KEGG pathway [67]. The *p*-value (cut-off 0.05) was used to detect the enrichment of GO terms and KEGG pathways.

## 5. Conclusions

In this study, miRNAs were found to be involved in regulating the fading of ornamental crabapple flower petals. A total of 247 miRNAs, including 230 known and 17 novel miRNAs, were identified using transcriptome and small RNA sequencing. Combined with degradome sequencing, we identified 194 miRNAs targeting 1641 target genes, forming 3971 putative miRNA–target pairs. The results of differentially expressed miRNA analysis showed that 52 DEMs targeted 494 target genes, among which 20 targeted 38 genes that may play an important role in flower fading. Through the miRNA–target regulatory network, it was found that miRNAs participate in the fading of flower petals by inhibiting anthocyanin synthesis via targeting transcription factors and key structural genes. It is also possible to inhibit anthocyanin transport from the cytoplasm to the vacuole by targeting the ABC transporter gene and accelerate anthocyanin degradome by targeting the peroxidase gene, which participates in the fading of petals. This study provides a novel approach to revealing the fading mechanism of ornamental crabapple petals and provides a reference for other plants exhibiting this fading phenomenon.

## Figures and Tables

**Table 1:** Sample data filtration statistics.

Sample	Total Reads	Low Quality	n% > 10%	With PloyA/T/G/C	5′ Adapter Contamine	3′ Adapter Null or Insert Null	Clean Reads
S1-1	11,130,851 (100.00%)	32,789 (0.29%)	284 (0.00%)	39,316 (0.35%)	22,268 (0.20%)	35,053 (0.31%)	11,001,141 (98.83%)
S1-2	11,551,119 (100.00%)	23,426 (0.20%)	264 (0.00%)	50,861 (0.44%)	20,191 (0.17%)	30,744 (0.27%)	11,425,633 (98.91%)
S1-3	11,120,356 (100.00%)	19,925 (0.18%)	288 (0.00%)	51,742 (0.47%)	19,573 (0.18%)	21,715 (0.20%)	11,007,113 (98.98%)
S2-1	15,250,003 (100.00%)	26,070 (0.17%)	2 (0.00%)	71,565 (0.47%)	37,295 (0.24%)	34,932 (0.23%)	15,080,139 (98.89%)
S2-2	10,378,914 (100.00%)	29,734 (0.29%)	0 (0.00%)	34,616 (0.33%)	21,127 (0.20%)	30,120 (0.29%)	10,263,317 (98.89%)
S2-3	13,167,261 (100.00%)	26,657 (0.20%)	2 (0.00%)	46,509 (0.35%)	29,992 (0.23%)	41,940 (0.32%)	13,022,161 (98.90%)
S3-1	12,471,933 (100.00%)	24,675 (0.20%)	323 (0.00%)	44,652 (0.36%)	31,989 (0.26%)	52,167 (0.42%)	12,318,127 (98.77%)
S3-2	10,377,813 (100.00%)	19,107 (0.18%)	103 (0.00%)	31,320 (0.30%)	22,521 (0.22%)	18,331 (0.18%)	10,286,431 (99.12%)
S3-3	12,481,753 (100.00%)	24,407 (0.20%)	295 (0.00%)	35,189 (0.28%)	36,021 (0.29%)	34,849 (0.28%)	12,350,992 (98.95%)

**Table 2:** Length number of sRNAs after filtering.

Sample	Total Reads	Total Bases (bp)	Uniq Reads	Uniq Bases (bp)
S1-1	10,326,285 (93.87%)	240,011,935	3,333,822	78,023,779
S1-2	10,764,689 (94.22%)	254,703,959	3,537,426	83,697,298
S1-3	10,577,850 (96.1%)	250,378,594	3,815,470	90,254,243
S2-1	14,317,555 (94.94%)	335,306,569	4,395,347	103,251,091
S2-2	9,750,218 (95%)	225,212,083	3,253,872	75,919,353
S2-3	11,812,488 (90.71%)	276,884,741	3,748,589	88,095,359
S3-1	10,224,011 (83%)	240,660,054	3,294,154	77,441,845
S3-2	8,870,908 (86.24%)	206,017,726	3,020,900	70,589,599
S3-3	10,762,409 (87.14%)	249,820,642	3,557,890	83,165,290

**Table 3:** Results of comparison between sRNA and reference genome.

Sample	Total Reads	Mapped sRNA	“+” Mapped sRNA	“−” Mapped sRNA
S1-1	10,326,285	8,655,944 (83.82%)	6,055,395 (58.64%)	2,600,549 (25.18%)
S1-2	10,764,689	8,755,122 (81.33%)	6,058,875 (56.28%)	2,696,247 (25.05%)
S1-3	10,577,850	8,720,375 (82.44%)	5,900,539 (55.78%)	2,819,836 (26.66%)
S2-1	14,317,555	11,813,538 (82.51%)	7,911,805 (55.26%)	3,901,733 (27.25%)
S2-2	9,750,218	8,242,418 (84.54%)	5,799,124 (59.48%)	2,443,294 (25.06%)
S2-3	11,812,488	9,944,221 (84.18%)	6,958,961 (58.91%)	2,985,260 (25.27%)
S3-1	10224011	8,646,339 (84.57%)	6,089,558 (59.56%)	2,556,781 (25.01%)
S3-2	8,870,908	7,482,395 (84.35%)	5,284,165 (59.57%)	2,198,230 (24.78%)
S3-3	10,762,409	8,991,361 (83.54%)	6,282,950 (58.38%)	2,708,411 (25.17%)

**Table 4:** Predicted miRNA and comparison of sRNA in each sample.

Types	Novel		Known		Mapped Uniq sRNA	Mapped Total sRNA
	miRNA	Hairpin	Mature	Hairpin		
Total	17	17	230	177	3614	2,946,473
S1-1	12	12	199	159	418	203,349
S1-2	12	12	195	151	349	168,439
S1-3	12	12	185	149	353	185,315
S2-1	15	15	208	168	425	308,377
S2-2	12	12	208	163	403	417,892
S2-3	14	14	206	162	428	417,377
S3-1	13	13	200	163	396	411,555
S3-2	12	12	215	171	404	397,709
S3-3	17	17	211	168	438	436,460

Note: (1) Mapped mature: predicted maturity. (2) Mapped hairpin: precursors that are predicted. (3) Mapped unique sRNA: the type of sRNA that is mapped against the precursor. (4) Mapped total sRNA: the number of sRNAs that are mapped against precursors.

**Table 5:** Sequencing results of degradome sequencing.

Types	DDSF (Number)	DDSF (Ratio)
Raw reads	22,232,259	/
Unique raw reads	8,699,925	/
reads < 15 nt after removing 3′ adaptors	577,231	2.60%
mappable reads	21,655,028	97.40%
Unique reads < 15 nt after removing 3′ adaptor	39,218	0.45%
Unique mappable reads	8,660,707	99.55%
Mapped reads	13,072,434	58.80%
Unique mapped reads	4,997,426	57.44%
Number of input transcripts	44,677	/
Number of covered transcripts	29,591	66.23%

DDSF, degradome sequencing of flowers.

## Data Availability

All the data are shown in the main manuscript, Supplementary Materials, and https://figshare.com/articles/dataset/The_T-plot_of_all_the_miRNA-target_pairs/23592387, accessed on 28 June 2023.

## Supplementary Materials

The following supporting information can be downloaded at https://www.mdpi.com/article/10.3390/ijms241411384/s1.

## References

[1] Han M.L., Yin J., Zhao Y.H., Sun X.W., Meng J.X., Zhou J., Shen T., Li H.H., Zhang F. (2020). How the Color Fades from *Malus halliana* Flowers: Transcriptome Sequencing and DNA Methylation Analysis. Front. Plant Sci..

[2] Sheng L., Xia W., Zang S., Zeng Y., Yuan X., Ning G., Zhang S., Feng L. (2018). Transcriptome-sequencing analyses reveal putative genes related to flower color variation in Chinese *Rosa rugosa*. Acta Physiol. Plant..

[3] Guo L., Wang Y., Da Silva J.A.T., Fan Y., Yu X. (2019). Transcriptome and chemical analysis reveal putative genes involved in flower color change in Paeonia ‘Coral Sunset’. Plant Physiol. Biochem..

[4] Yue Y., Liu J., Shi T., Chen M., Li Y., Du J., Jiang H., Yang X., Hu H., Wang L. (2019). Integrating Transcriptomic and GC-MS Metabolomic Analysis to Characterize Color and Aroma Formation during Tepal Development in *Lycoris longituba*. Plants.

[5] Casimiro-Soriguer I., Narbona E., Buide M.L., Del Valle J.C., Whittall J.B. (2016). Transcriptome and Biochemical Analysis of a Flower Color Polymorphism in *Silene littorea* (Caryophyllaceae). Front. Plant Sci..

[6] Wang Y., Zhou L.-J., Wang Y., Geng Z., Ding B., Jiang J., Chen S., Chen F. (2022). An R2R3-MYB transcription factor CmMYB21 represses anthocyanin biosynthesis in color fading petals of chrysanthemum. Sci. Hortic..

[7] Napoli C., Lemieux C., Jorgensen R. (1990). Introduction of a Chimeric Chalcone Synthase Gene into Petunia Results in Reversible Co-Suppression of Homologous Genes in trans. Plant Cell.

[8] Blokland R.V., Geest N.V.D., Mol J.N.M., Kooter J.M. (1994). Transgene-mediated suppression of chalcone synthase expression in Petunia-Hybrida results from an increase in RNA turnover. Plant J..

[9] Bashandy H., Pietiäinen M., Carvalho E., Lim K.-J., Elomaa P., Martens S., Teeri T.H. (2015). Anthocyanin biosynthesis in gerbera cultivar ‘Estelle’ and its acyanic sport ‘Ivory’. Planta.

[10] Sun J., Wu Y., Shi M., Zhao D., Tao J. (2020). Isolation of PlANS and PlDFR genes from herbaceous peony (*Paeonia lactiflora* Pall.) and its functional characterization in Arabidopsis and tobacco. Plant Cell Tissue Organ Cult..

[11] Nakatsuka T., Mishibaa K.-I., Abe Y., Kubota A., Kakizaki Y., Yamamura S., Nishihara M. (2008). Flower color modification of gentian plants by RNAi-mediated gene silencing. Plant Biotechnol..

[12] Nakatsuka T., Nishihara M., Mishiba K., Yamamura S. (2005). Two different mutations are involved in the formation of white-flowered gentian plants. Plant Sci..

[13] Alfenito M.R., Souer E., Goodman C.D., Buell R., Mol J., Koes R., Walbot V. (1998). Functional complementation of anthocyanin sequestration in the vacuole by widely divergent glutathione S-transferases. Plant Cell.

[14] Sasaki N., Nishizaki Y., Uchida Y., Wakamatsu E., Umemoto N., Momose M., Okamura M., Yoshida H., Yamaguchi M., Nakayama M. (2012). Identification of the glutathione S-transferase gene responsible for flower color intensity in carnations. Plant Biotechnol..

[15] Vilperte V., Boehm R., Debener T. (2021). A highly mutable GST is essential for bract colouration in *Euphorbia pulcherrima* Willd. Ex Klotsch. BMC Genom..

[16] Schaart J.G., Dubos C., De La Fuente I.R., Van Houwelingen A.M.M.L., De Vos R.C.H., Jonker H.H., Xu W., Routaboul J.-M., Lepiniec L., Bovy A.G. (2013). Identification and characterization of MYB-bHLH-WD40 regulatory complexes controlling proanthocyanidin biosynthesis in strawberry (*Fragaria* × *ananassa*) fruits. New Phytol..

[17] Ramsay N.A., Glover B.J. (2005). MYB-bHLH-WD40 protein complex and the evolution of cellular diversity. Trends Plant Sci..

[18] Gonzalez A., Zhao M., Leavitt J.M., Lloyd A.M. (2008). Regulation of the anthocyanin biosynthetic pathway by the TTG1/bHLH/Myb transcriptional complex in Arabidopsis seedlings. Plant J..

[19] Albert N.W., Lewis D.H., Zhang H., Schwinn K.E., Jameson P.E., Davies K.M. (2011). Members of an R2R3-MYB transcription factor family in Petunia are developmentally and environmentally regulated to control complex floral and vegetative pigmentation patterning. Plant J..

[20] Huang D., Wang X., Tang Z., Yuan Y., Xu Y., He J., Jiang X., Peng S.-A., Li L., Butelli E. (2018). Subfunctionalization of the Ruby2–Ruby1 gene cluster during the domestication of citrus. Nat. Plants.

[21] Cavallini E., Matus J.T., Finezzo L., Zenoni S., Loyola R., Guzzo F., Schlechter R., Ageorges A., Arce-Johnson P., Tornielli G.B. (2015). The Phenylpropanoid Pathway Is Controlled at Different Branches by a Set of R2R3-MYB C2 Repressors in Grapevine. Plant Physiol..

[22] Jun J.H., Liu C.G., Xiao X.R., Dixon R.A. (2015). The Transcriptional Repressor MYB2 Regulates BOTH Spatial and Temporal Patterns of Proanthocyandin and Anthocyanin Pigmentation in *Medicago truncatula*. Plant Cell.

[23] Xu H.F., Wang N., Liu J.X., Qu C.Z., Wang Y.C., Jiang S.H., Lu N.L., Wang D.Y., Zhang Z.Y., Chen X.S. (2017). The molecular mechanism underlying anthocyanin metabolism in apple using the MdMYB16 and MdbHLH33 genes. Plant Mol. Biol..

[24] Vaknin H., Bar-Akiva A., Ovadia R., Nissim-Levi A., Forer I., Weiss D., Oren-Shamir M. (2005). Active anthocyanin degradation in *Brunfelsia calycina* (yesterday-today-tomorrow) flowers. Planta.

[25] Oren-Shamir M. (2009). Does anthocyanin degradation play a significant role in determining pigment concentration in plants?. Plant Sci..

[26] Gou J.-Y., Felippes F.F., Liu C.-J., Weigel D., Wang J.-W. (2011). Negative Regulation of Anthocyanin Biosynthesis in Arabidopsis by a miR156-Targeted SPL Transcription Factor. Plant Cell.

[27] Zhao D., Xia X., Wei M., Sun J., Meng J., Tao J. (2017). Overexpression of herbaceous peony miR156e-3p improves anthocyanin accumulation in transgenic *Arabidopsis thaliana* lateral branches. 3 Biotech..

[28] Jie Z., Hao C., Ji T., Jie Z., Yuncong Y. (2020). The role of miR156a in the coloring of different Malus petals. J. Beijing Univ. Agric..

[29] Tian T., Liu Y., Yan H., You Q., Yi X., Du Z., Xu W., Su Z. (2017). agriGO v2.0: A GO analysis toolkit for the agricultural community, 2017 update. Nucleic Acids Res..

[30] Yang F., Cai J., Yang Y., Liu Z. (2013). Overexpression of microRNA828 reduces anthocyanin accumulation in Arabidopsis. Plant Cell Tissue Organ Cult..

[31] Li Z.Q., Liu W.J., Chen Q.J., Zhang S.H., Mei Z.X., Yu L., Wang C., Mao Z.Q., Chen Z.J., Chen X.S. (2023). Mdm-miR858 targets MdMYB9 and MdMYBPA1 to participate anthocyanin biosynthesis in red-fleshed apple. Plant J..

[32] Yu H.P., Song C.N., Jia Q.D., Wang C., Li F., Nicholas K.K., Zhang X.Y., Fang J.G. (2011). Computational identification of microRNAs in apple expressed sequence tags and validation of their precise sequences by miR-RACE. Physiol. Plant.

[33] Ye K.Y., Chen Y., Hu X.W., Guo J.C. (2013). Computational identification of microRNAs and their targets in apple. Genes Genom..

[34] Xia R., Zhu H., An Y.Q., Beers E.P., Liu Z.R. (2012). Apple miRNAs and tasiRNAs with novel regulatory networks. Genome Biol..

[35] Hutvagner G., Simard M.J. (2008). Argonaute proteins: Key players in RNA silencing. Nat. Rev. Mol. Cell Biol..

[36] Wu L., Zhang Q., Zhou H., Ni F., Wu X., Qi Y. (2009). Rice MicroRNA Effector Complexes and Targets. Plant Cell.

[37] Han M., Yang C., Zhou J., Zhu J., Meng J., Shen T., Xin Z., Li H. (2020). Analysis of flavonoids and anthocyanin biosynthesis-related genes expression reveals the mechanism of petal color fading of *Malus hupehensis* (Rosaceae). Braz. J. Bot..

[38] Die J.V., Jones R.W., Ogden E.L., Ehlenfeldt M.K., Rowland L.J. (2020). Characterization and Analysis of Anthocyanin-Related Genes in Wild-Type Blueberry and the Pink-Fruited Mutant Cultivar ‘Pink Lemonade’: New Insights into Anthocyanin Biosynthesis. Agronomy.

[39] Niu S.S., Xu C.J., Zhang W.S., Zhang B., Li X., Lin-Wang K., Ferguson I.B., Allan A.C., Chen K.S. (2010). Coordinated regulation of anthocyanin biosynthesis in Chinese bayberry (*Myrica rubra*) fruit by a R2R3 MYB transcription factor. Planta.

[40] Li Y.Q., Shan X.T., Zhou L.D., Gao R.F., Yang S., Wang S.C., Wang L., Gao X. (2019). The R2R3-MYB Factor FhMYB5 from Freesia hybrida Contributes to the Regulation of Anthocyanin and Proanthocyanidin in Biosynthesis. Front. Plant Sci..

[41] Wang Y., Wang Y., Song Z., Zhang H. (2016). Repression of MYBL2 by Both microRNA858a and HY5 Leads to the Activation of Anthocyanin Biosynthetic Pathway in Arabidopsis. Mol. Plant.

[42] Zhou B., Leng J., Ma Y., Fan P., Li Y., Yan H., Xu Q. (2020). BrmiR828 TargetsBrPAP1,BrMYB82, andBrTAS4Involved in the Light Induced Anthocyanin Biosynthetic Pathway in *Brassica rapa*. Int. J. Mol. Sci..

[43] Ding T.Y., Tomes S., Gleave A.P., Zhang H.T., Dare A.P., Plunkett B., Espley R.V., Luo Z.W., Zhang R.P., Allan A.C. (2022). microRNA172 targets APETALA2 to regulate flavonoid biosynthesis in apple (*Malus domestica*). Hortic. Res..

[44] Luo X.N., Luo S., Fu Y.Q., Kong C., Wang K., Sun D.Y., Li M.C., Yan Z.G., Shi Q.Q., Zhang Y.L. (2022). Genome-Wide Identification and Comparative Profiling of MicroRNAs Reveal Flavonoid Biosynthesis in Two Contrasting Flower Color Cultivars of Tree Peony. Front. Plant Sci..

[45] Roy S., Tripathi A.M., Yadav A., Mishra P., Nautiyal C.S. (2016). Identification and Expression Analyses of miRNAs from Two Contrasting Flower Color Cultivars of Canna by Deep Sequencing. PLoS ONE.

[46] Zhao D., Wei M., Shi M., Hao Z., Tao J. (2017). Identification and comparative profiling of miRNAs in herbaceous peony (*Paeonia lactiflora* Pall.) with red/yellow bicoloured flowers. Sci. Rep..

[47] Hu Y., Cheng H., Zhang Y., Zhang J., Niu S., Wang X., Li W., Zhang J., Yao Y. (2021). The MdMYB16/MdMYB1-miR7125-MdCCR module regulates the homeostasis between anthocyanin and lignin biosynthesis during light induction in apple. New Phytol..

[48] Yan X.J., Liu J., Kim H., Liu B.G., Huang X., Yang Z.C., Lin Y.C.J., Chen H., Yang C.M., Wang J.P. (2019). CAD1 and CCR2 protein complex formation in monolignol biosynthesis in Populus trichocarpa. New Phytol..

[49] Zhao J., Dixon R.A. (2010). The ‘ins’ and ‘outs’ of flavonoid transport. Trends Plant Sci..

[50] Debeaujon I., Peeters A.J.M., Leon-Kloosterziel K.M., Koornneef M. (2001). The TRANSPARENT TESTA12 gene of Arabidopsis encodes a multidrug secondary transporter-like protein required for flavonoid sequestration in vacuoles of the seed coat endothelium. Plant Cell.

[51] Marinova K., Pourcel L., Weder B., Schwarz M., Barron D., Routaboul J.M., Debeaujon I., Klein M. (2007). The Arabidopsis MATE transporter TT12 acts as a vacuolar flavonoid/H+-antiporter active in proanthocyanidin-accumulating cells of the seed coat. Plant Cell.

[52] Dixon D.P., Edwards R. (2010). Roles for Stress-inducible Lambda Glutathione Transferases in Flavonoid Metabolism in Plants as Identified by Ligand Fishing. J. Biol. Chem..

[53] Passamonti S., Cocolo A., Braidot E., Petrussa E., Peresson C., Medic N., Macri F., Vianello A. (2005). Characterization of electrogenic bromosulfophthalein transport in carnation petal microsomes and its inhibition by antibodies against bilitranslocase. FEBS J..

[54] Behrens C.E., Smith K.E., Iancu C.V., Choe J.Y., Dean J.V. (2019). Transport of Anthocyanins and other Flavonoids by the Arabidopsis ATP-Binding Cassette Transporter AtABCC2. Sci. Rep..

[55] Francisco R.M., Regalado A., Ageorges A., Burla B.J., Bassin B., Eisenach C., Zarrouk O., Vialet S., Marlin T., Chaves M.M. (2013). ABCC1, an ATP Binding Cassette Protein from Grape Berry, Transports Anthocyanidin 3-O-Glucosides. Plant Cell.

[56] Hasselkus E.R. (1985). Crabapple: Their Evaluation/Selection. MALUS Int. Ornam. Crabapple Soc. Bull..

[57] Ye Y., Wang J., Ni Z., Meng X., Feng Y., Yang Z., Xu L.-A. (2020). Small RNA and degradome sequencing reveal roles of miRNAs in strobilus development in masson pine (*Pinus massoniana*). Ind. Crops Prod..

[58] Wang J., Xu M., Li Z., Ye Y., Rong H., Xu L.-A. (2018). Tamarix microRNA Profiling Reveals New Insight into Salt Tolerance. Forests.

[59] Zhang L., Hu J., Han X., Li J., Gao Y., Richards C.M., Zhang C., Tian Y., Liu G., Gul H. (2019). A high-quality apple genome assembly reveals the association of a retrotransposon and red fruit colour. Nat. Commun..

[60] Langmead B., Trapnell C., Pop M., Salzberg S.L. (2009). Ultrafast and memory-efficient alignment of short DNA sequences to the human genome. Genome Biol..

[61] Kuang Z., Wang Y., Li L., Yang X.Z. (2019). miRDeep-P2: Accurate and fast analysis of the microRNA transcriptome in plants. Bioinformatics.

[62] Friedlander M.R., Mackowiak S.D., Li N., Chen W., Rajewsky N. (2012). miRDeep2 accurately identifies known and hundreds of novel microRNA genes in seven animal clades. Nucleic Acids Res..

[63] Axtell M.J., Meyers B.C. (2018). Revisiting Criteria for Plant MicroRNA Annotation in the Era of Big Data. Plant Cell.

[64] Kozomara A., Birgaoanu M., Griffiths-Jones S. (2018). miRBase: From microRNA sequences to function. Nucleic Acids Res..

[65] Huang B., Rong H., Ye Y., Ni Z., Xu M., Zhang W., Xu L.A. (2020). Transcriptomic analysis of flower color variation in the ornamental crabapple (*Malus* spp.) half-sib family through Illumina and PacBio Sequel sequencing. Plant Physiol. Biochem..

[66] Addo-Quaye C., Miller W., Axtell M.J. (2009). CleaveLand: A pipeline for using degradome data to find cleaved small RNA targets. Bioinformatics.

[67] Xie C., Mao X., Huang J., Ding Y., Wu J., Dong S., Kong L., Gao G., Li C.-Y., Wei L. (2011). KOBAS 2.0: A web server for annotation and identification of enriched pathways and diseases. Nucleic Acids Res..

