# Supplementary figures and images for: Small RNA and Degradome Sequencing Reveal Roles of miRNAs in the Petal Color Fading of Malus Crabapple
Source: Int J Mol Sci. 2023 Jul 13;24(14):11384. doi: 10.3390/ijms241411384 (PMC10379340; doi:10.3390/ijms241411384)

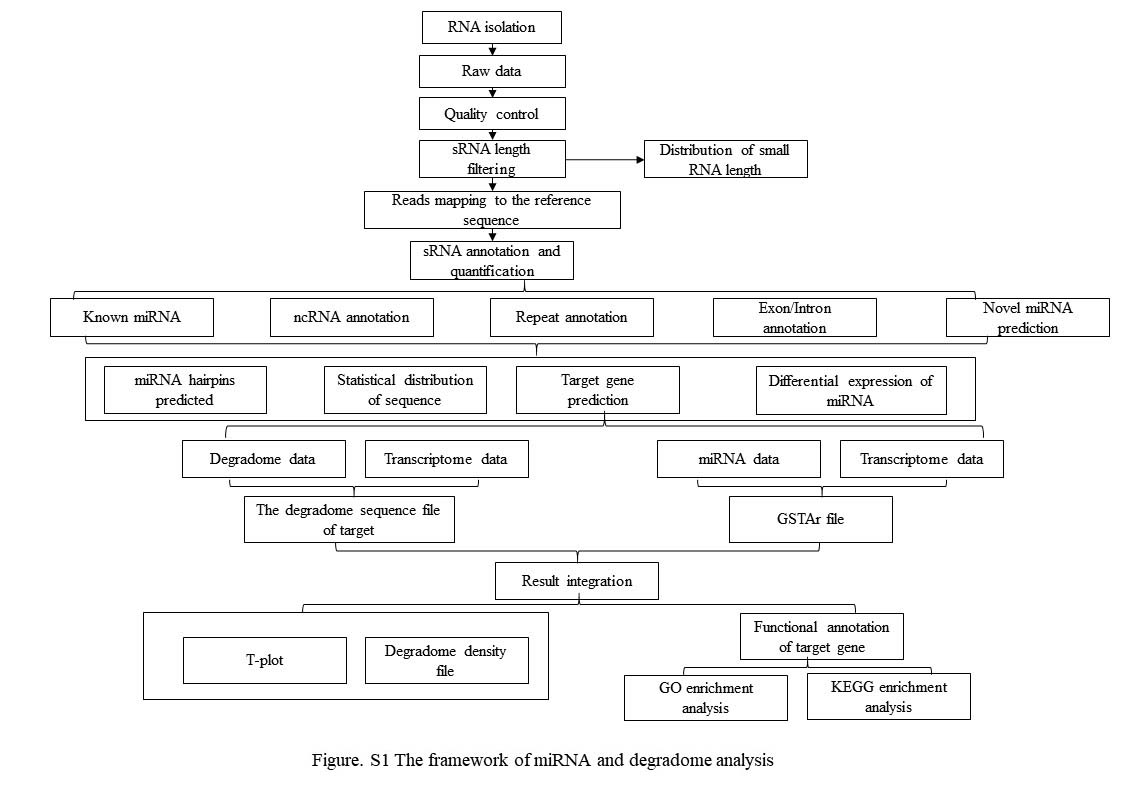

Supplement: Supplementary file 1 [file ijms-24-11384-s001.zip › Figure S1.jpg]
